# Involvement of Ceramides in Non-Alcoholic Fatty Liver Disease (NAFLD) Atherosclerosis (ATS) Development: Mechanisms and Therapeutic Targets

**DOI:** 10.3390/diagnostics11112053

**Published:** 2021-11-05

**Authors:** Daniela Maria Tanase, Evelina Maria Gosav, Daniela Petrov, Alina Ecaterina Jucan, Cristina Mihaela Lacatusu, Mariana Floria, Claudia Cristina Tarniceriu, Claudia Florida Costea, Manuela Ciocoiu, Ciprian Rezus

**Affiliations:** 1 Department of Internal Medicine, “Grigore T. Popa” University of Medicine and Pharmacy, 700115 Iasi, Romania; tanasedm@gmail.com (D.M.T.); dr.evelinagosav@gmail.com (E.M.G.); ciprianrezus@yahoo.com (C.R.); 2 Internal Medicine Clinic, “Sf. Spiridon” County Clinical Emergency Hospital Iasi, 700111 Iasi, Romania; 3 Department of Rheumatology and Physiotherapy, “Grigore T. Popa” University of Medicine and Pharmacy, 700115 Iasi, Romania; danielapetrovdoc@gmail.com; 4 I Rheumatology Clinic, Clinical Rehabilitation Hospital, 700661 Iasi, Romania; 5 Department of Gastroenterology, “Grigore T. Popa” University of Medicine and Pharmacy, 700115 Iasi, Romania; ghiata.alina.ecaterina@gmail.com; 6 Institute of Gastroenterology and Hepatology, “Sf. Spiridon” County Clinical Emergency Hospital, 700111 Iasi, Romania; 7 Unit of Diabetes, Nutrition and Metabolic Diseases, “Grigore T. Popa” University of Medicine and Pharmacy, 700115 Iasi, Romania; cristina.lacatusu@umfiasi.ro; 8 Clinical Center of Diabetes, Nutrition and Metabolic Diseases, “Sf. Spiridon” County Clinical Emergency Hospital, 700111 Iasi, Romania; 9 Internal Medicine Clinic, Emergency Military Clinical Hospital Iasi, 700483 Iasi, Romania; 10 Department of Morpho-Functional Sciences I, Discipline of Anatomy, “Grigore T. Popa” University of Medicine and Pharmacy, 700115 Iasi, Romania; cristinaghib@yahoo.com; 11 Hematology Clinic, “Sf. Spiridon” County Clinical Emergency Hospital, 700111 Iasi, Romania; 12 Department of Ophthalmology, Faculty of Medicine, “Grigore T. Popa” University of Medicine and Pharmacy, 700115 Iasi, Romania; costea10@yahoo.com; 13 2nd Ophthalmology Clinic, “Prof. Dr. Nicolae Oblu” Emergency Clinical Hospital, 700309 Iasi, Romania; 14 Department of Pathophysiology, Faculty of Medicine, “Grigore T. Popa” University of Medicine and Pharmacy, 700115 Iasi, Romania; mciocoiu2003@yahoo.com

**Keywords:** non-alcoholic fatty liver disease, NAFLD, ceramide, sphingolipids, SL, atherosclerosis, ATS, biomarkers, therapeutic targets

## Abstract

Non-alcoholic fatty liver disease (NAFLD) and atherosclerosis (ATS) are worldwide known diseases with increased incidence and prevalence. These two are driven and are interconnected by multiple oxidative and metabolic functions such as lipotoxicity. A gamut of evidence suggests that sphingolipids (SL), such as ceramides, account for much of the tissue damage. Although in humans they are proving to be accurate biomarkers of adverse cardiovascular disease outcomes and NAFLD progression, in rodents, pharmacological inhibition or depletion of enzymes driving de novo ceramide synthesis prevents the development of metabolic driven diseases such as diabetes, ATS, and hepatic steatosis. In this narrative review, we discuss the pathways which generate the ceramide synthesis, the potential use of circulating ceramides as novel biomarkers in the development and progression of ATS and related diseases, and their potential use as therapeutic targets in NAFDL-ATS development which can further provide new clues in this field.

## 1. Introduction

Non-alcoholic fatty liver disease (NAFLD) is a chronic worldwide known liver disease, recently known as metabolic associated fatty liver disease (MAFLD), which subsumes hepatic steatosis and steatohepatitis (NASH), and leads to many different hepatic and extrahepatic complications. MAFLD was recently proposed by experts as a more appropriate overarching term, and its diagnosis should be based on the presence of metabolic dysfunction, not the absence of other conditions [1].

Presently in the fast-food and sedentary lifestyle era, the rate of obesity has increased, which is one of the main conditions that can lead to NAFLD, insulin resistance (IR), type 2 diabetes (T2DM), and other metabolic disorders. The development of NAFLD is based on various processes that result from ratio synthesis impairment, inflow of free fatty acids (FFAs), export of triglycerides (TG), and disruption of lipogenesis. Lipid accumulation in the liver, along with fibrosis induces cell injury, which may lead to cirrhosis onset and its many complications [2,3]. Patients that associate NAFLD, obesity and IR have a high risk to develop cardiovascular disease (CVD), being additionally, connected by diverse risk factors such as dyslipidemia, which promotes blood vessel wall lipid accumulation [4]. Myriad research has demonstrated that NAFLD is associated with a greater risk of CVD development, and that the presence of CVD dictates outcomes in patients with NAFLD more frequently, and to a greater extent than does the progression of liver disease. Notably, a part of these resulted FFAs is esterified to diacylglycerol (DAG) and triacylglycerol (TAG), while a smaller part is involved in the sphingolipid’s (SL) biosynthesis of ceramides. In the last decade, researchers brought valuable information that highlight the involvement of ceramides as important contributors to the inflammatory and metabolic pathways in obesity, hepatic steatosis and in cardiovascular pathologies, such as ATS [5,6]. A better knowledge of the pathophysiological links between NAFLD and ATS, could offer new insights into possible effective interventional modalities benefiting both disorders.

In this review, we describe the metabolism of SL and ceramides, the involvement of ceramides in the development of NAFLD-ATS association, and mostly, discuss their potential as biomarkers and therapeutic targets in NAFLD and ATS.

## 2. Ceramides

Ceramides are bioactive lipid intermediates and members of the sphingolipid family that take part in the formation of the lipid bilayer of the cell membrane. Although ceramide synthesis occurs in actually all organs in the body, the principal site for ceramide production is the liver [7]. In cultured cells and isolated tissues, ceramides perturb mitochondrial function, block fuel usage, disrupt vasodilatation and promote apoptosis [8,9]. They are generated through three different modalities: de novo synthesis, a sphingomyelinases (SMases) pathway (catabolic), and a salvage pathway [10].

The de novo pathway of ceramides, considered the precursor of the most complex sphingolipids, is the most predominant and best characterized pathway for ceramide accumulation. It occurs on the cytosolic surface of the endoplasmic reticulum (ER) and depends on the availability of fatty acids [11]. A large spectrum of inflammatory stimuli (e.g., tumor necrosis factor-α [TNF-α], interferons), hormonal signals, ionizing radiations, inducers of cells (differentiation, apoptosis and growth suppression), promote the ceramide biosynthesis. Additionally, this pathway can be induced by metabolic loading with serine, oxidative stress, and oxidized low-density lipoproteins (ox-LDL) [11,12]. De novo sphingolipid synthesis requires several enzymatic steps performed by enzymes such as ceramide synthases (CerS), serine palmitoyl-CoA transferase (SPT), 3-ketosphinganine reductase (KSR) and dihydroceramide desaturase (DES), which are continuously explored for their pharmacological potential as targets in certain metabolic diseases [13]. The ceramide biosynthesis pathway starts with the condensation of palmitoyl-CoA and serine, to produce 3-ketosphinganine, a reaction that is catalyzed by SPT [14]. 3-ketosphinganine is then reduced to sphinganine via KSR. In the end, N-acylation of sphinganine by CerS1-S6, produces dihydroceramide which is afterwards converted into ceramide via dihydroceramide desaturase 1 (Des1) [15]. Therefore, ceramides are synthesized by a family of six CerS, each isoform displaying specificities for the chain length of the added acyl-CoA [16,17,18,19,20,21,22,23,24,25,26,27], and possess different properties depending on their chain length (Table 1).

The complexity of sphingolipid biosynthesis follows the formation of ceramide on the membranes of the ER and continues in the Golgi apparatus, their transport to Golgi is facilitated by the action of the ceramide transfer protein. Here, they serve as precursors for the synthesis of complex SL, such as sphingomyelin (SM) and glucosylceramides [28].

The second major reaction that results in ceramide production is SM hydrolysis. In this pathway ceramides are generated via hydrolysis of the choline head group from SM, by the action of either acid or neutral sphingomyelinase (N-SMases) enzymes. In addition to activation of de novo ceramide synthesis, inflammation up-regulates the activity of N-SMases. Accumulation of ceramides in this compartment of cell contribute to ER stress following to death cell and furthermore, lipotoxicity [29]. However, there are not a lot of studies that demonstrates the direct role of ceramides in these processes. Ceramides can cause cellular dysfunction, ultimately cell death and block the activation of protein kinase B (PKB), also called Akt, contributing so to metabolic impairment. In their in vitro study, Charusia et al. [30], demonstrated that deletion of gene delta 4-desaturase sphingolipid 1 (Degs1) which encoded Des1, reduced adiposity, decreased steatosis and hepatic gluconeogenesis, and improved insulin sensitivity. More than that, suppression of Des1 improves mitochondrial activity by reducing lipid accumulation and thus, decreases the chance of developing ATS [30]. This evidence highlights the involvement of ceramides in diseases such as NAFLD and ATS, and brings new ideas to the possible use of ceramides as therapeutic targets that could be beneficial in both disorders.

Additionally, ceramide can be formed by the salvage or the so-called “recycling” pathway, which is the third major pathway, in which the sphingosine derived from SL catabolism serves as a substrate for ceramide re-generation [31]. This process takes place in the late endosomes and lysosomes, and involves several key enzymes such as SMases, ceramidases and dihydroceramide synthases. The ceramide accumulation resulted from the catabolism of complex sphingolipids that are broken down eventually into sphingosine, are reused through reacylation to produce ceramide. The phosphorylation of sphingosine-by-sphingosine kinases (SphK1 and SphK2) produces sphingosine 1-phosphaet (S1P), a molecule involved in angiogenesis, proliferation, migration, transformation, and inflammation of cells. This SL turnover pathway has been estimated to contribute from 50% up to 90% of the ceramide biosynthesis [31,32]. The salvage pathway is not only subject to regulation, but it also modulates the formation of ceramide and subsequent ceramide-dependent cellular signals [33].

All three pathways described, individually or coordinately, contribute to ceramide synthesis, leading to the generation of ceramide signaling and subsequent modulation of several cellular responses which are involved in NAFLD-ATS development.

### Ceramides in NAFLD-ATS Development

ATS, an important well-known disease, develops progressively as a result of inflammation and lipid accumulation. This term is composed by two elements, one of them is “atherosis” which means aggregation of fats, and “sclerosis”-accumulation of fibrosis in the main inner layers of arteries [34]. Hyperlipidemia is the bridge between NAFLD and ATS events, resulting in the release of low-density lipoproteins (LDL) in circulation and the initiation of the oxidative processes. LDL are associated with a high cardiovascular risk by deposition into vessels walls as they implicitly participate to the atheroma plaque formation. Although lipidomic analysis of human atherosclerotic plaques and liver pattern of NAFLD patient revealed different phospholipid composition, a common key finding was ceramides [35]. These have harmful effects on hepatic metabolism via lipotoxicity, which is induced especially by the excess of saturated fatty acids, TG, contributing therefore to NAFLD onset and progression [36,37,38]. They also are involved in endothelial dysfunction and can influence the mobilization of various inflammatory cytokines, metalloproteinases, making the atheroma plaque vulnerable and more susceptible to rupture or erosion [39]. As mentioned, ceramide species can induce endothelial dysfunction and increase vascular permeability by promoting oxidative stress, inflammation and by reducing the production of nitric oxide (NO) [40]. Therefore, ceramides are important regulators of tissue metabolism and play vital roles in pathophysiological processes with implications in cardiometabolic disease such as ATS [41], inflammation, obesity, NAFLD [42], pancreatic ß-cell dysfunction, T2DM [43], and cancer [44].

Regarding their involvement in atherogenesis, they participate by two mechanisms: one directly on the vascular wall through transcytosis of ox-LDL, and second through insulin sensitivity and dyslipidemia [44]. In different circumstances, data shows that ox-LDL is present in the atherosclerotic plaque, and that increased blood concentration especially in patients with NAFLD, mediates activation of LacCer synthase and thus, the development of ATS [45,46]. Ox-LDL can lead to arterial endothelium damage by inducing cell proliferation and can establish the secretion of TNFα, a result of uptake in macrophages and accumulation in blood flow. More than that, proliferation of arterial smooth muscle cell, as a main step of development of ATS is generated by the ox-LDL, which activates the oxygen-sensitive signaling pathway [47]. All those complex processes along with ceramide accumulation and, subsequently modification of intracellular signaling pathways contribute to inflammatory signals and reformation of intracellular organelles and ER function [44,48]. In development of fatty acid oxidation, mitochondria play a major role in production of different reactive oxygen species (ROS). Supersaturation of hepatocytes with fatty acids promotes liver oxidative stress by activating mitochondrial activity and ROS production [49]. Cell damage enhances inflammation, and as long as inflammatory cytokines conduct to increased ceramide production, in return ceramides through feedback mechanisms lead to different production of cytokines, forming a vicious circle [36].

**Figure 1:**
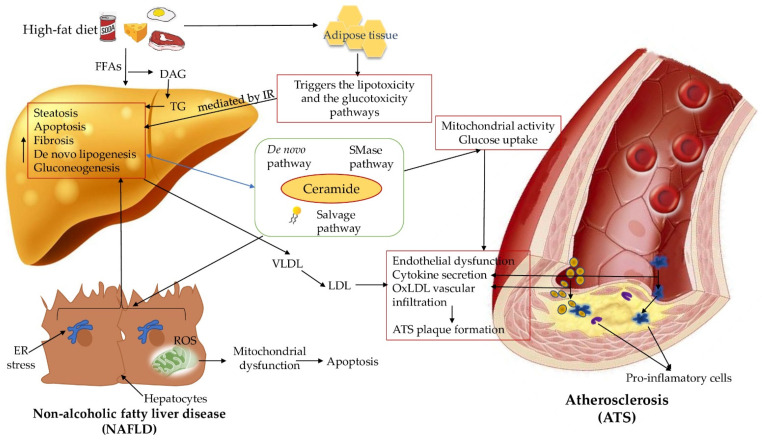
Ceramides as mediators behind NAFLD-ATS pathogenesis development. insulin resistance (IR); tumor necrosis factor-α (TNF-α); interleukin-6 (IL-6); free fatty acids (FFAs); diacylglycerol (DAG); triglycerides (TG); very-low-density lipoproteins (VLDL); low-density lipoproteins (LDL); oxidized low-density lipoproteins (ox-LDL); sphingomyelinases (SMase); endoplasmic reticulum (ER); reactive oxygen species (ROS).

Overall, the human body has more than 500 lipid species identified, each and own with its particular role in metabolic disease development [50]. Along with oxysterols-species ceramide, contribute to hepatic steatosis, development of artery plaque and regulate expression of pro-inflammatory genes linked to plaque instability [51,52]. These elements point out the role of ceramides as key specimen in this vicious circle that includes almost the whole lipid metabolism, oxidative and inflammation cascades [53,54]. Although ceramides are involved in ATS as both membrane components and lipid mediators, their precise role in atherosclerosis remains elusive. However, due to their particular characteristics and the significant role in inflammation, IR, and oxidative stress, it is seen that ceramides act as mediators and contribute to the pathogenesis of both diseases. However, further scientific discoveries are needed to elucidate the exact mechanisms behind the link that connects NAFLD with ATS development and progression (Figure 1), which will grant new data that could promote the use of ceramide as potential novel biomarkers or therapeutic targets in metabolic diseases.

## 3. Ceramides as Potential Biomarkers in Atherosclerosis Related Diseases

Traditional biomarkers of atherosclerotic cardiovascular disease (ACVD), such as blood levels of lipoproteins, brain-type natriuretic peptides (BNPs) and C-reactive protein (CRP) are not universally applicable even if they possess well-researched prognostic and diagnostic value [55]. Thus, researchers continue to explore new potential biomarkers for ACVD. Sipos et al. [56] highlighted in their review four promising new representative biomarkers which have a meaningful role in detecting inflammatory processes in CVD: soluble suppression of tumorigenicity 2 (sST2) [57], heart-type fatty acid binding protein (H-FABP) [58], growth differentiation factor (GDF-15) [59], and soluble urokinase-type plasminogen activator receptor (suPAR) [60]. These markers are of great interest as they can reflect pathological diseases with reference to ATS and related diseases, provide additional prognostic information about CVD risk in populations [61], or achieve knowledge about the response to certain therapies. However, more clinical trials are necessary for the optimal use of these biomarkers, and the research must be continued to obtain more accurate information.

Recent studies have highlighted the potential use of circulating ceramides as novel biomarkers in the development and progression of CVD, as they can have more predictive value than LDL cholesterol [62,63,64]. Their role as novel biomarkers is also investigated in cardiovascular related diseases, such as type-2 diabetes, IR, or obesity [65,66,67]. Ceramides can not only assess the risk of cardiac events but also can provide additional information regarding the cardiovascular risk in patient populations with certain predisposing conditions [68,69,70]. For that reason, ceramide risk scores have been formulated and have been found to be associated with an increased incidence of future cardiovascular events [71]. The findings in cited works, suggest the benefit and added value of analyzing certain plasma ceramide species when assessing the risk of CVD events and CVD death.

In their trial, Meeusen et al. [72] investigated the predictive utility of ceramides and the ceramide risk score in a cohort study of United States patients referred for coronary angiography at Mayo Clinic Cardiovascular Laboratory Medicine. The ceramide risk score combined the values of the most commonly involved ceramides in ATS processes: N-palmitoyl-sphingosine [Cer(16:0)], N-stearoylsphingosine [Cer(18:0)], and N-nervonoyl-sphingosine [Cer(24:1)], and N-lignoceroyl-sphingosine [Cer(24:0)] into a single score. After a 4-year follow-up, they exposed that Cer(16:0), Cer(18:0), Cer(24:1) have predictive power in coronary artery dysfunction which includes myocardial infarction, cardiomyopathy, heart failure, and stroke. Additionally, 3 plasma ceramides and their ratios to a fourth ceramide Cer (24:0) have all significantly predictive role for major adverse cardiovascular events (MACE) and death by any cause [72]. Patients with high risks must be monitored to adjust therapy and to avoid development of complications. Therefore, there is a need for new potential biomarkers such as lipid species, especially ceramide (d18:1/16:0), that potentially qualify prognostic markers for ACVD. 

Another study conducted by Bodini et al. [73] reported that ceramides can be a promising source of diagnostic and prognostic biomarkers in cardiovascular disease by identifying people at low cardiovascular risk, without coronary ATS. They evaluated which plasma lipids can significantly contribute to improve the identification of subjects at minimal risk and, also, re-estimated the minimal risk tool (reMRT) by adding the lipids from 13 among ceramides, TG, and sphingomyelins to the baseline model. The sensitivity analysis confirmed that plasma lipidomics can be a useful and exploitable tool to identify subjects without coronary ATS, hence reducing unnecessary further testing in normal individuals.

For the first time, ceramide risk scores were used in secondary [74], and in primary prevention [75] for cardiovascular disease such as CAD. The notorious randomized trial (PREDIMED trial—Prevention with Mediterranean Diet) [76], and the prospective community-based cohort study conducted by Vasile et al. [77] strengthened the idea that ceramide risk scores have predictive value for cardiovascular events and that they can be applied particularly in patients at intermediate risk. In their research, Poss et al. [78], using random forest (RF) and the least absolute shrinkage and selection operator (LASSO) regression approaches for variable reduction and selection, created new sphingolipid-based risk scores. An RF-generated sphingolipid-inclusive CAD (RF-SIC) risk score outperformed (cardiac event risk test 1) CERT1 (AUC = 0.67) and conventional CVD risk biomarkers including LDL-C (AUC = 0.69). Compared with the CERT1 originally developed by Zora Biosciences and validated in multiple prospective clinical studies [64], the sphingolipid-inclusive (SIC) risk score registered superiority in predicting CAD. The components included in the SIC risk score are: dihydro-cer(d18:0/18:0), cer(d18:1/18:0), cer(d18:1/22:0), cer(d18:1/24:0), dihydro-SM(d18:0/24:1), SM(d18:1/24:0), SM(d18:1/18:0), and sphingosine [78,79].

Hilvo et al. [64] investigated the prognostic role of CERT2 by adding certain phosphatidylcholines (PCs) to the ceramide test score CERT1 in prediction of CVD events in patients with atherosclerotic coronary heart disease. The new ceramide test score, defined as CERT2, was developed in the WECAC study (The Western Norway Coronary Angiography Cohort) and validated in the LIPID (The Long-Term Intervention with Pravastatin in Ischaemic Disease) and KAROLA (Langzeiterfolge der Kardiologischen Anschlussheilbehandlung) trials. In the present study, the authors demonstrated that the CERT2 score provided relevant prognostic value and accurate information and showed that improved prognostics can be obtained by combining CERT2 with other biomarkers, such as hsTnT (high-sensitivity troponin-T). Therefore, a single blood-based prognostic analysis demonstrated the strong association of CERT2 and CERT2-TnT with CVD death and CVD events. In addition to the aforementioned study, Hilvo et al. [80] investigated the performance of CERT2 in a large primary prevention cohort study compared to classical lipid biomarkers, and its ability to stratify risk along with total cholesterol (TC), which is the lipid biomarker currently used in the SCORE risk charts. CERT2 highlighted the added value of phospholipids for risk score and for identification of very high-risk individuals in primary prevention. Regarding to the previous study, Vasile et al. [81] highlighted that in comparison with CERT1, CERT2 led to a better reclassification of indices, and seems to have additive value and be at least comparable to SCORE. Nonetheless, more validation studies are needed before implementing CERT2 as a routine analysis for primary prevention.

As a main idea, the sources cited above have pointed a causative role for ceramides in the development and progression of ATS and its complications, and the ceramide risk scores have been found to be associated with an increased incidence of future cardiovascular events, thus, ceramides can be attractive potential predictive and/or prognostic biomarkers ACVD [82].

## 4. Ceramides as Potential Therapeutic Targets

A myriad of research, including some clinical trials such as NCT02133144 [83], or NCT02211612 [84], have displayed the impact of high saturated fat consumption on human organism. For that reason, lipid excess leads to increased intrahepatic TG accumulation, lipolysis, and harmful plasma ceramides formation. Given their common role in the onset and progression of both metabolic diseases NAFLD and ATS, ceramides continue to be researched as potential therapeutic targets. By this novel approach inhibition of ceramide pathway could alleviate hepatic inflammation precursors, oxidative cell secretion, circulating LDL aggregation and foam cell formation, benefiting both disease [85].

### 4.1. Myriocin

Thermozymocidin also known as Myriocin is an irreversible and high affinity inhibitor of SPT, an enzyme involved in up-regulation in de novo synthesis of ceramide that has therapeutic potential against diabetes, ATS and hepatic steatosis by decresing ceramide levels [86]. In humans treatment with Myriocin did not changed the amount of LDL particles or TG, but rather it changed the composition of LDL particles, especially the concentration of SM [87]. Additionally, Yang et al. [88] demonstrated the efficacy of Myriocin in NAFLD severity by decreasing the levels of plasma ceramides. They examined three groups of mice: control group, rats fed with standard diet for 16 weeks, and the third group formed by rats with high-fat diet (HFD) + Myriocin 0.3 mg/kg on alternate days from the 8 week to 16 week by gavage. Rats fed with HFD increased in body weight, while those with Myriocin decreased in body weight, and their hepatic levels of LC-3II and p62 were inverted to normal levels. They observed decreasing of LC-3 II/I ratio and increasing of p62 at 12, 24 and 48 h post FFAs incubation, while in Myriocin cells the level of LC-3 II/I was restored to normal at 12 and 24 h, at 48 h for p62 [88].

Another study analyzed the effect of Myriocin on 4–6 weeks rats and the importance of lowering ceramides in development of NAFLD. By dividing also, into same three groups, they noted that the serum ceramide content were remarkable higher in those with high-fat diet, and that Myriocin treatment reduced ceramide content, liver TG and improved the level of inflammation, the expression of TNF-α, and significantly attenuated hepatic fibrosis, the expression of p-JNK/JNK (p-Jun N-terminal kinase/Jun N-terminal kinase), cytochrome c, and Bax, a multitude of markers involved in apoptotic processes. Hence therapy with Myriocin may help reduce the risk of developing NAFLD and later NASH [89].

### 4.2. CerS Inhibitors

With inflammation as a main link, additional therapies such as methotrexate, PCSK9 inhibitors or interleukin-1b inhibition, may be new strategies for treating cardiovascular diseases [90]. Moreover, several enzymes of the sphingolipid synthesis have already been tested as potential drug targets as their inhibition has been shown to decrease ATS [74]. There are six mammalian CerS (CerS1-6) known, each being codified on a different chromosome [91]. Each CerS are distributed differently in tissue. A model of CerS2-null mice, generates disruption of different cellular pathways and biochemical processes with decrease of C22–C24 ceramides [91]. Since ceramides, are implicated in a lot of diseases, CerS might potentially be targets for therapeutic direction in various pathologies [85].

Subsequent research identified that not only deficiency in CerS can modulate ceramide levels, inhibition of several CerS also affect the serum levels of ceramides, one of them is Fumonisin B1 (FB1), a CerS inhibitor produced by *Fusarium moniliforme* which contains an aminopentol backbone with two hydroxyl groups esterified with tricarballylic acids [92]. CerS inhibition by FB1 triggers a “perfect storm” of alterations in structural and signaling sphingolipids such as: reduced formation of dihydroceramides and ceramides. Practically, this compound inhibits sphinganine and acyl-CoA [93]. Because of the potential hepato-renal toxicity, the clinical use of fumonisin B1 is limited [94]. The affection caused by FB1 can be called “sphingolipidoses”, and the authors attention users about the consequences of CerS inhibition with future precautions when investigating other naturally occurring and synthetic compounds [95].

Dysregulation of the sphingolipid pathway has been described in several inflammatory and immune-mediated diseases such as systemic lupus erythematosus (SLE) [96]. Data shows that SLE patients have higher plasma levels of total ceramides, including C16:0, C18:1, C18:0, C20:1, C:20:0, C24:1, and 26:1 Cer species [97]. As ceramides are associated with apoptosis and inflammation which are pathways to ATS and CAD, SLE patients have a raised risk of disease development [98]. Thus, it is essential to find a treatment that would decrease the concentration of ceramides and somewhat all phenomena triggered by them, in order to decrease this risk. Evidence describes the role of S1P in NAFLD, IR and obesity development. There are two sphingosine kinase isoforms, SPHK1 and SPHK2, which synthesize S1P by phosphorylating sphingosine [99]. With this in mind, another inhibitor of CerS is Australia fungin FTY720 (Fingolimod), an FDA-approved drug for treatment of multiple sclerosis and an agonist of S1P receptors (S1PR) that inhibits CerS by noncompetitive inhibition of acyl-CoA [6,100]. He is also researched as a potent immunosuppressive agent currently in Phase III clinical trials for kidney transplantation [93]. In a recent article, oral administration of FTY720 in diet-induced animal model of NAFLD (DIAMOND), improved glucose tolerance, reduced steatosis and TG levels [101]. Aditionally, treatment with FTY720 reduced liver sphingolipid levels, including ceramides, monohexosylceramides, and sphingomyelins, especially the C16:0 and C24:1 species, as well as dihydro-S1P and S1P [100,101]. After 20 weeks of high-cholesterol diet to apolipoprotein E deficient mice, FTY720 drastically reduced atherosclerotic lesion volume (62.5%), most probably by uppressing the machinery involved in monocyte/macrophage emigration to atherosclerotic lesions. Given the fact that vascular S1P receptors stayed functional under Fingolimod, S1P agonists that selectively target the vasculature and not the immune system, may be promising new therapy against ATS [102].

On this note, a small part of the HDL population contains apolipoprotein M (ApoM), which is the main plasma carrier of the bioactive lipid mediator S1P. The majority of S1P is linked to ApoM and have an impact on the atherosclerotic process by regulation of adhesion molecule abundance, leukocyte-endothelial adhesion, and endothelial barrier. This axis also has proposed in recent years for ATS treatment [103,104]. A detailed scientometric research study which analyzed the use of S1P in age-related diseases, recommend evaluating the interconnection and unintended side effects of S1P, with the future possibility of discovering novel avenues for research and broaden the clinical value of S1P [105]. Interestingly, genetic deficiency of SphK2 but not SphK1, aggravates the formation of atherosclerotic lesions in mice with ApoE deficiency. Their results indicate that SphK2 may be a novel target for treating ATS, as it is required for autophagosome- and lysosome-mediated catabolism of intracellular lipid droplets to interfere with the development of ATS [106].

### 4.3. Des1 Inhibitors

Another caching therapeutic target is Des1, an enzyme that catalyzes the final step in the de novo synthesis of ceramide: the insertion of a C4 double bond into the precursor dihydroceramide to form ceramide [107]. The study reported by Chaurasia et al. [108] revealed that inducible genetic ablation of DES1, which prevents the conversion of dihydroceramides to ceramides during de novo sphingolipid synthesis, has substantial improvement in insulin sensitivity, prevention of pancreatic b-cell dysfunction, and resolution of liver fat, hepatocyte inflammation and fibrosis [108]. Likewise, animal studies pointed out that with the decline of DEGS1 (the gene encoding for Des1), de novo biosynthesis of ceramide is disturbed [109]. Development of inhibitors of Des1 in the ceramide pathway [110] offers exciting opportunities to reduce the cardiometabolic disease for a large number of suffering individuals, therefore, further research is needed in order to decipher the complex role of dihydroceramides in cell biology and reveal new therapeutic approaches.

### 4.4. MTP Inhibitors

Accretion microsomal triglyceride transfer proteins are important in regulating the secretion, synthesis of ceramides and are directly involved in the sphingolipid transport to the plasma by setting release of beta-lipoproteins [111]. Apo-B-containing lipoproteins (B-lps) are a type of lipoproteins involved in transport of different types of lipids inclusive ceramides [112]. The loss of this protein in humans is called abetalipoproteinemia (ABL), an autosomal recessive disorder with an estimated frequency of <1 in 1,000,000 [111,113]. In an experimental animal study, authors objected that MTP is involved in ceramide and sphingomyelin secretion, but not in their synthesis, also MTP might regulate plasma ceramide and sphingomyelin levels by transferring these lipids to B-lps in the liver and intestine, and facilitate their secretion [111,113]. They compared a mouse model of ABL, with deleted MTP gene in the liver and small intestine and controls group. Mice plasma concentrations of TG, phospholipid, cholesterol, and ceramide were reduced by >90% compared with control mice. Plasma sphingomyelin levels were reduced by ~73% in the MTP-deficient mice, and all levels of ceramides decreased [114]. The selective inhibition of MTP triglyceride transfer activity may reduce hyperlipidemia while protecting the hepatic parenchyma from excess lipid accumulation [115]. Disrupting the formation of ceramides by ABL in patients or MTP-deficient mice leads to a decrease in plasma ceramides concentrations and implicitly to a decrease in the formation of atheroma plaque [99]. Additionally, treatment with the MTP inhibitor led to reversal of hyperlipidemia in atherosclerotic mice, and beneficial changes in the composition and the inflammatory state of the plaque [116]. MTP inhibitor may serve as a therapeutic target especially in those with less therapeutic effect with current treatments, such as statins.

### 4.5. ASMase Inhibitors

Enzyme such as acid sphingomyelinase (aSMase) catalyzes the hydrolysis of sphingomyelin (ASMase) to ceramide, mediates redox signaling in coronary arterial endothelial cells [117].

Palmitic acid (PA) and lipopolysaccaharide (LPS) play a major role in this processes by stimulating ASM and up-regulates pro-inflammatory cytokines [118,119]. High sphingolipid levels are linked with plaque instability through contribution of SMases in different inflamatory pathways [120]. The aSMase and neutral SMase (nSMase), especially the type 2-neutral SMase (nSMase2—a cloned N-SMase isoform), are activated in vascular cells by inflammatory agents and may contribute to endothelial activation and inflammation [30].

To elucidate the role of nSMase2 in atherogenesis, researchers explored a genetic double mutant mouse model deficient in both nSMase2 activity and ApoE deficient and a pharmacological model of long-term inhibition of nSMase2 by GW4869 in ApoE deficient mice [121]. The results show that the genetic deficiency of nSMase2 or its pharmacological inhibition by GW4869, significantly reduced the size of atherosclerotic areas, and the accumulation of macrophages, by 68% in mice treated with GW4869 compared to 49% control mice [122] Additionally, the deficiency or inhibition of nSMase2 activity resulted in a significant decrease in plasma ceramide levels, particularly in 24:1, 22:0, and 24:0 ceramide levels [122,123]. The inhibition of nSMase2 suppress inflammation by two mechanisms: a short time process implicating Nrf2 (nuclear factor [erythroid-derived 2]-like 2 or) that plays as an anti-inflammatory response, and long-term action, by decreasing the production of pro-inflammatory ceramides [124]. The pharmacological or genetic inhibition of nSMase2 was not associated with plasma lipoprotein changes, but with the direct effect on the arterial wall [122].

Lu et al. [122] noticed in their metabolic study that both amitriptyline and GW4869 reduced glucose, lipids, and IR. Amitriptyline inhibits ATS through modulation of sphingolipid metabolism [125]. Given to mice with LDL receptor-deficient that contain high amounts of SFFA, such as PA, a high-fat diet, they observed an increased development of systemic inflammation, activation of Toll-like receptors in macrophages and cytokines, with subsequently ATS [122]. Collectively, this research pointed that amitriptyline inhibited NASH and ATS through modulation of sphingolipid metabolism in rodents, indicating that sphingolipid metabolism in macrophages plays a crucial role in the linkage of NASH and ATS [121,122].

Imipramine, another tricyclic antidepressant, significantly decreased the total ceramide concentrations, phospho-p38, phospho-JNK and steatosis levels in ethanol-fed mice [126]. ASMase inhibitors may be considered to be a therapeutic target for alcohol-induced hepatic steatosis and stress kinases activation.

### 4.6. Lip-C6

Ceramide have also a negative impact on the balance of energy homeostasis, including the inhibition of the energy-sensor adenosine monophosphate activated kinase (AMPK) phosphorylation and different transcription agents such as Nrf2 [127]. The general effects of ceramides depends on specific chain lengths, a few years ago started the preclinical development of the Ceramide NanoLiposome (CNL), as an anticancer drug for the initial indication of hepatocellular carcinoma (HCC). The CNL short-chain C6-ceramide (Lip-C6), can actually exercise anti-inflammatory and anti-lipogenesis effects [128]. Zanleri et al. [129] studied the effects of non-apoptotic systemic doses of the cell permeable ceramide Lip-C6 on AMPK-and Nrf2-dependent oxidative stress [129]. The results showed that Lip-C6 is not as metabolically active, treatment induced a strong phosphorylation of AMPK in methionine-choline deficient (MCD)-fed mice, but overall reversed the imbalance in hepatic phosphatidylcholines and diacylglycerides species, energy/metabolic depletion and raised protective anti-oxidant signaling pathways, most likely by restoring homeostatic lipid function [129].

### 4.7. Glucagon-like Peptide-1 (GLP-1)

Liraglutide a GLP-1 receptor agonist, (GLP-1R), beyond its action on T2DM it has directly effects on hepatocytes, limit ER stress and has anti-steatotic effects [130,131]. Methionine-choline deficient (MCD) diet rodents have a higher development of steatosis and inflammation, with elevated concentration of ceramides especially CER16, CER24, compared to the MCD-liraglutide-treated mice, who showed decreased levels of plasma lipids [132]. Different gene expression of Sptlc2, cerS4, and cerS6 were down-regulated by liraglutide, emphasizing the effect of liraglutide on sphingolipid metabolism and mainly on evolution of NAFLD [133]. Another role of GLP-1R is to prevent accumulation of ceramide in the cardiac progenitor cells, and the subsequently development of various cardiovascular pathologies, such as ATS [134,135]. GLP-1-based therapies appear to provide beneficial effects against ATS and NAFLD; however, additional randomized data will be required to arrive at conclusive evidence.

### 4.8. Intestinal FXR/SMPD3 Axis

Another therapeutic target that should be considered is the intestinal farnesoid X receptor (FXR), which is a ligand-activated nuclear receptor significantly activated in patients with hypercholesteremia and mice fed a high-cholesterol diet (HCD) [136]. Recent studies performed on HCD-rodents showed that intestinal FXR deficiency or direct FXR inhibition (via treatment with the FXR antagonist glycoursodeoxycholic acid [GUDCA]), decreased ATS and reduced the levels of circulating ceramides and cholesterol, all these results suggesting that intestinal FXR modulates intestinal ceramide production [137]. Furthermore, in this study they identified sphingomyelin phosphodiesterase 3 (SMPD3), as an FXR target gene which can regulate cholesterol catabolism by modulating the hepatic CYP7A1 activity [137]. Hepatic CYP7A1 represents the rate-limiting enzyme in the classical bile acid synthesis pathway, and, also, the key enzyme involved in cholesterol catabolism, which can potentiate ATS [138]. Additionally, they reported that fibroblast growth factor 15/19 (FGF15/19) binds to FGF receptor 4 (FGFR4) to suppress hepatic expression of CYP7A1 [138]. This study showed that intestinal FXR/SMPD3 inhibition could be considered to be a therapeutic target for ATS treatment by lowering the circulating ceramide level and by protecting against metabolic diseases including obesity, IR, and fatty liver [138].

### 4.9. Other Therapeutic Approaches

Velázquez et al. [139] revealed that administration of high-fat-high-fructose (HF-HFr) diet causes hypertriglyceridemia and hepatic lipid deposition, but not inflammation, ER stress or oxidative stress. Moreover, liver ceramides were reported to be increased in NASH, but not in simple steatosis in humans [139]. They compared four groups: first the control group, second HF-HFr, third high-fat diet containing caffeine (CAF), fourth high-fat diet containing a green coffee extract providing 0.18 g of caffeine/kg of diet, and 10% fructose in the drinking water. The HF-HFr diet significantly reduced the levels of Cer 14:0 and Cer 16:0 and fat liver accumulation [136]. It is known by now that regular coffee consumption is significantly associated with a reduced risk of NAFLD and of liver fibrosis [140]. More epidemiological studies are needed to validate coffee consumption as an essential preventive measure in hepatic steatosis [141]. Besides coffee consumption, a dietary change based on increasing intake of fruit and vegetables in parallel with decreasing consumption of refined carbohydrates or fat, may reduce serum concentration of ceramides. Targeting dietary patterns, showed the importance of healthy diet in lowering the concentration of plasma ceramides and decrease in cardiovascular risk [142].

Mathews et al. [143] discovered that 8 week of free-living fruit and vegetables diet can lower ceramide concentration. All 36 subject were divided into three groups: fruit and vegetables, low refined carbohydrates and low fat. They discovered that majority of ceramides, especially C24:0 a known insulin signaling modulator and the most abundant ceramide in circulation, diminished by week 5, up to 48% in the fruit and vegetables plus low fat group. Additionally, C24:0 was correlated with pro-inflammatory cytokines such as IFN-γ (Interferon-γ) or interleukin-10 (IL-10). In addition to the decrease in ceramide levels, a decrease in waist circumference, systolic boold presure and circulating cholesterol was, also, noticed [143]. All data collected from the study, point out the importance of a health diet concerning the diminishing of ceramide content and augment clinical biomarkers in metabolic and cardiovascular disorders.

The traditional Mediterranean diet (MedDiet) may have the potential to lessen the injurios effect associated with elevated baseline plasma ceramide concentrations on CVD risk [144]. The effect of MedDiet was studied in a PREDIMED trial consisted of 980 participants including 230 incident cases of CVD and 787 randomly selected participants at baseline (including 37 overlapping cases), tracked up to 7.4 years. They observed how MedDiet intervention modified the damaging effect of higher ceramide concentrations on CVD risk. Consumption of MedDiet may directly influence ceramide biosynthesis by changing circulating FFAs composition through modifying dietary fat intake, decreasing saturated fat entrance, by improving monounsaturated and polyunsaturated fat intakes, and by modulating de novo lipogenesis upon improvement in dietary carbohydrate quality [76].

Alpha-mangostin and aSMase/ceramide pathway may modulate NO production by regulating reactive oxyge species (ROS) production and improve endothelial dysfunction [145]. Hence, another pharmacological approach described by Jiang et al. [146] involves alpha-mangostin, a naturally compound detached from various parts of the mangosteen tree that inhibits elevated aSMase/ceramide pathway. They figured out that treatment with alpha-mangostin ameliorates endothelial dysfunction in vivo and in vitro, through inhibition of the aSMase/ceramide course. The vascular dysfunction in the diabetic group was partly regained by the alpha-mangostin treatment, accompanied by lowered aSMase activity and ceramide content in normal mice [146].

Nutrition takes a central role in NAFLD development, micronutrients such as electrolytes, minerals, vitamins, and carotenoids, are needed to sustain physiologic functions. The lock micronutrients have been reported as crucial in NAFLD progression [147]. Sangineto et al. [148] explored the probable beneficial effects of dietary supplementation with FLINAX, an innovative composition of nutraceuticals (vitamin E, vitamin D3, olive dry-extract, cinnamon dry-extract and fish oil) in a NAFLD model characterized by oxidative stress and mitochondrial function damaged. The Flinax group significantly presented lower hepatic fat accumulation compared to non-supplemented. The administration of Flinax protected the liver, reducing important lipoperoxidation markers, crucial enzymes in fatty acids oxidation (FAO), tangled in acyl-CoA formation such as carnitine palmitoyltransferase 1A (CPT1A) and carnitine palmitoyltransferase 2A (CPT2) [149].

Despite the therapeutic potential of sphingolipids (Table 2), the ability to develop this class of compounds as active pharmaceutical ingredients has been hampered by issues of solubility and delivery. Beyond these technical hurdles, significant challenges in completing the necessary preclinical studies to support regulatory review are necessary for commercialization.

**Table 2:** Deletion of specific genes, enzymes or supplementation with a specific diet helps to decrease the concentration of ceramides. sphingomyelin (SM); sphingosine kinase 1 (SPHK1); sphingosine kinase 2 (SPHK2); ceramide synthase 2 (CerS2); ceramide synthase 5 (CerS5); ceramide synthase 6 (CerS6); fruit and vegetables (FRUVED); low refined carbohydrates (LRC); ceramide (Cer); triglycerides (TG); alanine-aminotransferase (ALT); aspartate transaminase (AST); dihydroceramide desaturase (DES); Adenosine 5’-triphosphate (ATP); control group (Ctrl); fatty acid elongase 6 (Elovl6); ceramide synthase 1 (CERS1); carnitine palmitoyltransferase 1A (CPT1A); carnitine palmitoyltransferase (CPT2); serine palmitoyltransferase long chain base subunit 2 (Sptlc2).

Main Focus	Species	Outcomes	Year	Ref.
Fumonisine B1	Mice	60% reduction of hepatic SM levels (*p* < 0.05), increase expression of hepatic SPT (*p* < 0.01); SPHK 1 maximal at the lowest dose of 0.75 mg/kg (*p* < 0.05), expression of SPHK2 not affected;	2006	[92]
Elovl6	Mice	Reduced: Ceramide(d18:1/18:0)(0.63, *p* < 0.001); ceramide (d18:2/18:0) (1.68, *p* < 0.05), oleate (C18:1n-9) and stearate (C18:0);	2020	[150]
CerS2	Mice	Reduced lipid accumulation, sphingomyelin levels ~50%, uptake in the liver, reduction in very long chain acyl ceramides, enzymatic activity-decreased;	2015	[151]
CerS6	Mice	Reduce C16:0 ceramides, serum insulin concentrations, protects from macrophage infiltration, activation of pro-inflammatory gene expression; improve glucose tolerance and insulin sensitivity; reduced adiposity and increased energy expenditure, (*p* < 0.05);	2014	[152]
Diet	Human	Ceramides C22:0, C24:1; C26:0 reduced-29%, (*p* < 0.05), C24:0 50%, (*p* < 0.01); at week 8 increase of C16:0 (*p* < 0.05);	2017	[76]
P053	Mice	5 mg/kg/day reduced C18 ceramide by 31%, (*p* < 0.01); Reduces whole-body fat mass and the weight of white adipose depots;	2018	[17]
GW4869	Mice	Decrease of: the atherosclerotic area, accumulation of macrophages by 68%; atherosclerotic lesions by 69% (*p* < 0.001), in plasma Cer24:1, Cer22:0, and Cer24:0, (*p* < 0.05), lipid accumulation by 68% (*p* < 0.01);	2018	[121]
CerS1	Mice	The sphingolipid content in heart, liver, and white adipose tissue—not affect, imprivement of liver glucose metabolism, 95% reduction in C18:0 ceramide;	2019	[153]
CerS5	Mice	Improves glucose tolerance, insulin sensitivity, reduces white adipose inflammation, In skeletal muscle without obvious decrease;	2019	[153]
Bortezomib	Mice	Increase hepatic CerS2 expression, protects from development of NAFLD, decreases weight gain, TG levels lower (*p* < 0.01);	2019	[154]
Exendin-4	Mice	Decrease lobular inflammation (*p* = 0.18), fibrosis stages (*p* = 0.24)	2019	[155]
DEGS1 gene	Mice	Decreased: Cer16:0–0.09, Cer18:0–0.1 (*p* < 0.001), whole-body insulin sensitivity-restored, selective insulin resistance-reversed in the liver (*p* < 0.001);	2019	[115]
Myriocin	Rats	Reduced: serum ceramide content reduced, (*p* < 0.05), hepatic triglyceride, ALT, AST, hepatic inflammation, amount of inflammatory cell; Bcl-2 expression restored, (*p* < 0.05);	2019	[87]
Fenretinide	Mice	Lowered: plaque area 50.8% (*p* < 0.05), plasma lipid levels by 20.1%, (*p* < 0.05), and plasma ceramides;	2020	[156]
Diet	Mice	Flinax reduced lipoperoxidation markers, hepatic fat accumulation restores complex I, III, V (ATP-synthase), lower peroxides levels, (*p* < 0.05), no difference in complex IV, and higher production of CPT1A and CPT2;	2021	[148]
Alpha-mangostin	Mice	Inhibits: ceramide content, and, Inhibites aSMase activity,	2021	[146]
Farnesoid X receptor	Mice	Lower: ceramide content, hepatic cholesterol levels, mRNA levels of Smpd3 elevates hepatic Cyp7a1 mRNA levels Repressed lesion areas in aortas and smaller atherosclerotic lesions;	2021	[120]
Liraglutide	Mice	Gene expression Sptlc2, cerS4, and cerS6 decreased; C16 and C24 accumulation was limited (*p* < 0.05); Unchanged: saturated fatty acid, phospholipids with long chains-reduced, phospholipids with very long chains	2021	[132]

## 5. Conclusions

It is known that patients with NAFLD have an elevated risk of cardiovascular events such as ATS, and a wide spectrum of evidence emphasized the role of the dyslipidemia as a disruption of the balance of hepatic lipid metabolism. NAFLD and ATS are interconnected by complex mechanisms that perturb the lipid homeostasis with subsequently dysfunctional sphingolipid metabolism. As observed, ceramides are the major contributors to the sphingolipid’s family, owning multiple pathophysiological functions. Various in vivo and in vitro studies support ceramides as new future biomarkers in NAFLD-ATS and their associated disease, and promote their introduction in risk scores for better disease diagnosis. Given their recent resonance in this field, they are also, currently being investigated as possible new therapeutic targets in metabolic diseases such as NAFLD and ATS. We hope that further larger clinical trial will emerge and provide clues behind the whole complex ceramide-system which will provide newer directions in NAFLD-ATS management.

## Figures and Tables

**Table 1:** The synthesis of ceramides is regulated by six ceramide synthases (CerS1–CerS6), and their expression profiles differ throughout the body. The CerS are differentially distributed in various tissues, presumably to meet the different physiological needs of each tissue; Acyl-CoA Specificity (Acetyl-Coenzyme A Specificity).

Ceramide Synthases (CerS)	Tissue Distribution	Acyl-CoA Specificity	The Biophysical Properties	Ref.
CerS 1	Brain Skeletal muscle	C18, C18:1	Play important roles in signaling and sphingolipid development; Promotes insulin resistance in humans;	[16,17]
CerS 2	Liver Kidney	C20, C22,C24, C24:1, C26	Play vital roles in postnatal liver development and physiology; Have major molecular roles in the maintenance of normal liver homeostasis;	[18,19]
CerS 3	Intestine Skin Testes	C18, C18:1,C20, C22, C24, C24:1	Maintain the water permeability barrier function; Involved in sperm formation and androgen production;	[20,21]
CerS 4	Heart Liver	C18, C20	Highly expressed in liver cancer tissues and can facilitate HCC (Hepatocellular Carcinoma) formation; Controls homeostatic epidermal barrier maintenance;	[22,23]
CerS 5	Lung Prostate Thymus Spleen Skeletal muscle	C14, C16, C18, C18:1	Contributes to the development of diet-induced obesity; Play roles in sphingosine salvage pathway signaling and in the response to cellular stress;	[24,25]
CerS 6	Lymph node Intestine	C14, C16, C18	May serve as biomarkers in determining the effectiveness of anticancer agents; C16:0 is a significant factor in the development of obesity and its related complications.	[26,27]
